# Study of parasitic fauna of three species of deer occurring in southeastern Mexico

**DOI:** 10.1007/s00436-025-08518-9

**Published:** 2025-06-30

**Authors:** Brenda Solórzano García, Gerardo Pérez Ponce de León

**Affiliations:** Laboratorio de Parasitología y Medicina de la Conservación, Escuela Nacional de Estudios Superiores, Mérida (ENES-Mérida, UNAM), Yucatán, México

**Keywords:** Deer, Filariid, Gastrointestinal parasites, Cross-species transmission, Health status

## Abstract

Three species of deer can be found in southeastern Mexico, the white-tailed deer (*Odocoileus virginanus*), the red brocket deer (*Mazama temama*), and the Yucatan brocket deer (*O. pandora*). Here, we applied a combination of non-invasive sampling, necropsy examinations, and molecular diagnostics to assess the endoparasite infections fauna of in wild and captive populations of these three deer species. We analyzed a total of 140 samples and found eleven taxa parasitizing these hosts, including two trematodes (*Paramphistomum* sp., an unidentified trematode), one anoplocephalid cestode, six nematodes (*Strongyloides* sp., two morphotypes of strongylid eggs, *Mammomonogamus* sp., *Setaria* sp. and an unidentified ascarid), and two protozoans (*Giardia intestinalis* and unidentified ciliates). The Yucatan brocket deer showed the highest percentage of infected samples (39.2%), followed by the white-tailed deer (38.7%). For the red brocket deer, only two samples were positive to two parasite taxa (20%). Captive populations showed the lowest parasite richness and percentage of infection. Some of the parasites reported here can pose potential risk for the health of deer populations, and along with habitat loss and poaching, could jeopardize the persistence of deer in the area. This study adds to the knowledge gap on the parasitic fauna in wild populations of these culturally and ecologically important species, particularly for the Yucatan brocket deer for which parasitological information is very scarce, and highlights the necessity to keep monitoring the health status of deer in this area.

## Introduction

The Yucatán Peninsula (YP) located in southeastern Mexico, is one of the most biodiverse areas in the country (Islebe et al. 2015). Giving its geographic location and geomorphologic characteristics, it presents a broad variety of climate regimens and different tropical forest ecosystems, harboring a high variety of flora and fauna (Torrescano-Valle and Folan 2015). In this geographic region, three of the five species of deer (Cervidae) found in Mexico occur, namely, the white-tailed deer (*Odocoileus virginianus*), the red brocket deer (*Mazama temama*), and the Yucatan brocket deer (*Odocoileus pandora*) (Gallina and Mandujano 2009). Although sympatric in most of their range within the YP (Weber 2014), these three species present different ecological requirements and show different tolerances to habitat disturbance (Reyna-Hurtado and Sanchez-Pinzón 2019). The white-tailed deer is the largest in size and the most plastic of the three; it has a wide diet breadth, and it has been frequently observed in modified habitats such as crops and secondary vegetation, as well as in proximity to human presence (Reyna-Hurtado and Sanchez-Pinzón 2019). The Yucatan brocket deer is a medium size cervid, whereas the red brocket deer is the smallest deer in Mexico; the red brocket deer shows a more specialized diet, hence its association with undisturbed tropical forests (Gallina-Tessaro et al. 2019b).

These three species of deer are the main items of subsistence hunting and sport hunting and are important components of local cultural traditions (Ramírez-Barajas and Calmé 2015; Pinkus-Rendón and Rodríguez-Balam 2020). Nonetheless, overexploitation has triggered the decline and even eradication of local populations of these species strongly affecting their conservation status (Weber 2014; Ramírez-Barajas and Calmé 2015; Burgos-Solis 2020). Moreover, in the last decades anthropogenic disturbances have continuously impacted the forest tracts of the YP, reducing the amount of natural forest, hence deer habitat (Rada et al. 2015). Consequently, habitat loss and fragmentation along with poaching constitute major threats for these species in the country (Gallina and Mandujano 2009).

Even though these species have great cultural value and constitute principal components of the local fauna, the parasites that occur in wild populations have been poorly studied in the country. Most of the available parasitological reports have focused on white-tailed deer and red brocket deer kept in captivity, with only a few studies presenting parasitological information of free-living individuals (e.g., Romero-Castañón et al. 2008; Mukul-Yerves et al. 2014; Pavón-Rocha et al. 2020) (Table 1), whereas no information is available on the parasitic fauna of the Yucatan brocket deer. Nonetheless, it has been acknowledged that parasitic infections and diseases represent potential risks for these species, especially when synergistic effects with other threats such as overhunting or habitat loss are taken into consideration (Gallina-Tessaro et al. 2019a; Zhu et al. 2021).

**Table 1:** Parasites reported for white-tailed deer and red brocket deer. Parasite names were included as reported in publications. *Bold indicates parasite taxa reported in Mexico

Host species	Parasite group	Parasite taxa	References
White-tailed deer (*Odocoileus virginianus*)	Protozoa	*Babesia odocoilei*, *Eimeriaspp***.***,*Giardia intestinalis*, *Isospora sp*.*, *Plasmodium odocoilei*, *Sarcocystis* sp., *Theileria cervi,* Toxoplasma gondii*	(Walker and Becklund 1970; Garnham and Kuttlerf 1980; Montes Pérez et al. 1998; Trout et al. 2003; Mukul-Yerves et al. 2014; Pavón-Rocha et al. 2020)
	Nematoda	*Ascarissp., *Ashworthius* sp., *Bunostomum sp., *Capillaria sp., *Chabertia ovina*, *Cooperia sp., *Dictyocaulus viviparus*, *Elaeophora schneideri*, *Eucyathostomum longesubulatum*, *E. webbi*, *Gongylonema pulchrum*, *Haemonchus sp., *H. contortus*, *Mammomonogamus sp., *Mazamastrongylus odocoilei*, *M. pursgloveri*., *Monodontus louisianensis*, *Necator* sp., *Nematodirus sp., *N. filicollis*, *N. odocoilei*, *Obeliscoides cuniculis*, *Odocoileostrongylus tenuis*, *Oesophagostomum cervi*, *O. venulosum*, *Ostertagia* spp. *O. mossi*, *O, odocoilei*, *Parascaris sp.,*Parelaphostrongylus tennis*, *Protostrongylus coburni*, *P. rufescens.*, S*. cervi*, *S. labiatopapillosa*, *S. yehi*, *Skrjabinema parva*, *Strongyloides sp., *S. papillosus*, *Teladorsagia circumcincta*, *Trichuris sp., *T. ovis*, *Trichostrongylus sp.,*T. axei*, *Wehrdikmansia cervipedis*	(Walker and Becklund 1970; Forrester et al. 1974; Montes Pérez et al. 1998; Carreno et al. 2001; Samuel et al. 2001; Romero-Castañón et al. 2008; Campbell and Vercauteren 2011; Mukul-Yerves et al. 2014; Salmorán-Gómez et al. 2019; Cottingham et al. 2022)
	Trematoda	*Dicrocoelium dendriticum*, *Fascioloides magna*, *Heterobilharzia americana*, *Paragonimus sp.**,***Paramphistomum liorchis*, *P. cervi	(Walker and Becklund 1970; Carreno et al. 2001; Romero-Castañón et al. 2008; Campbell and Vercauteren 2011; Salmorán-Gómez et al. 2019)
	Cestoda	*Echinococus granulosus*, *Moniezia sp., *M. benedeni*, *M. expansa*, *Taenia spp*., *Thysanosoma actinioies*	(Walker and Becklund 1970; Montes Pérez et al. 1998; Campbell and Vercauteren 2011)
Red brocket deer (*Mazama temama*)	Protozoa	*Eimeria sp.	(Salmorán-Gómez et al. 2019)
	Nematoda	*Ascaris sp., *Mammomonogamus sp*., Setaria bidentata*, *Strongyloides sp., *Trichuris sp.	(Mukul-Yerves et al. 2014; Gomez-Puerta and Mayor 2017; Salmorán-Gómez et al. 2019)
	Trematoda	*Paramphistomum cervi	(Romero-Castañón et al. 2008)
	Cestoda	*Taenia sp.	(Salmorán-Gómez et al. 2019)

In this study, we use a combination of non-invasive sampling, microscopic examinations, and molecular diagnostic techniques to assess the gastrointestinal parasites and filariid infections in wild and captive populations of white-tailed deer, red brocket deer, and Yucatan brocket deer within three regions across their distribution across the YP. Additionally, gastrointestinal parasite specimens were collected from an opportunistic necropsy performed on a hunted white-tailed deer individual. Finally, diagnosis was complemented with a molecular characterization to corroborate parasite species identity.

## Methods

### Sample collection

**Fig. 1:**
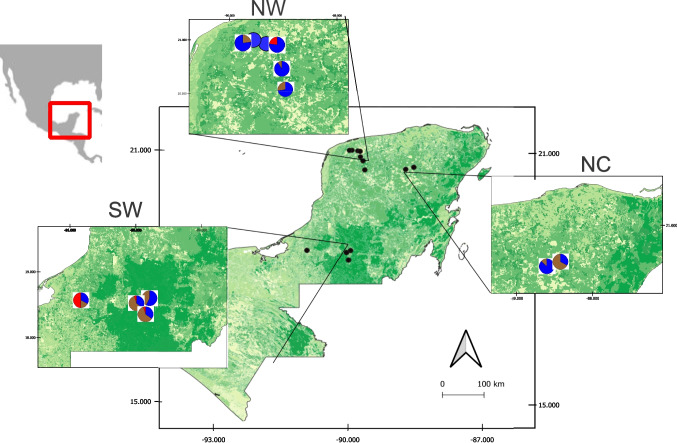
Collecting sites of the three species of deer across the Yucatan Peninsula (YP), southeastern Mexico. Pie charts indicate the proportion of samples collected from each host species: blue = white-tailed deer (*Odocoileus virginianus*), red = red brocket deer (*Mazama temama*), and brown = Yucatan brocket deer (*O. pandora*). NW = Northwestern region, NC = North-central region, SW = southwestern region

Collecting sites comprise several locations within three regions across the YP including a variety of tropical forest such as dry tropical forest in the Northwestern region (NW), medium semi-evergreen forest in the North-center region (NC), and medium evergreen forest in the southwestern region (SW) (Fig. 1). Deer fecal samples were collected opportunistically by identifying and following deer trails. Each trail was assumed to belong to one individual, thus only one sample per trail was collected to avoid duplicated samples from the same individual host. To ensure sample quality and reduce potential contamination, only fresh fecal samples were considered according to color, consistency, and the presence of fungi and top pellets were collected evading those in contact with the ground. In most locations, more than one species of deer was present; thus, host species identity was confirmed by molecular analysis (unpublished data). Samples from individuals in captivity were also collected from white-tailed deer (*N* = 22) and red brocket deer (*N* = 4) bred in two zoological gardens at Mérida city, Yucatán, i.e., the Zoológico Centenario and the Animaya zoo. Samples were collected in 50-ml vials, fixed with RNAlater buffer and kept at room temperature until processing.

### Morphological examinations

For the identification of gastrointestinal parasites, fecal samples were examined under direct light microscope (10 ×, 40 ×, 100 ×) using flotation in saturated sodium chloride solution and simple sedimentation techniques (Greiner and McIntosh 2009). Morphological identification of protozoan and helminth parasites was based on shape, size, and color. Each observed parasitic form, including cysts and different egg morphotypes was measured (length and width) and photographed to characterize its shape. Prevalence of infection was estimated for each parasite taxa (or morphotype) in each host species and in each region.

During the sampling period, we had the opportunity to examine the gastrointestinal tract of one white-tailed deer specimen that was hunted in a local community. Organs were longitudinally dissected, and tissue and content was filtered at 0.0049 in. sieve. The collected residuals were examined under the stereoscopic microscope looking for adult helminth parasites. The recovered parasite specimens were fixed and preserved in ethanol. For detailed morphological examinations, the recovered nematodes were cleared with alcohol–glycerol solution and observed using a Motic BA210 light microscope. En face view observations were made following the technique proposed by (Solórzano-García et al. 2024b) consisting of mounting the parasite inside a cut micropipette tip filled with commercial hair styling gel, attached to a microscope slide, and placing the cover slide on top to observe under the microscope.

### Molecular analysis

#### qPCR detection of filariid DNA

To assess the presence of filariid infections genomic DNA was extracted for each collected sample using the Quick‐DNA Fecal/Soil Microbe MiniPrep Kit (Zymo Research). Filariid DNA was screened in all samples by qPCR targeting a fragment of the 28S gene using the steps, primers and conditions specified in (Solórzano-García et al. 2024a). The qPCR screening was performed in a step-one RT-PCR system (Applied Biosystems). Negative and positive controls (from a previously extracted DNA from a filariid nematode) were included in each TaqMan cycle. To further corroborate filariid identity, qPCR positive samples were subjected to a standard PCR for the amplification of a fragment of the 12S gene, using the filariid specific primers and conditions (Laidoudi et al. 2020).

#### Molecular characterization of gastrointestinal parasites

Fecal samples microscopically classified as positive for *Giardia* sp. were subjected to a nested PCR to amplify a 130 bp fragment of the 18S ribosomal gene using the outer primers RH11 and RH4, and inner primers, GiarF–GiarR following published protocol (Gillhuber et al. 2013).

To corroborate the identity of the adult parasites recovered from the gastrointestinal tract, DNA was extracted from individual specimens following standardized protocols (Solórzano-García et al. 2015). The mitochondrial cytochrome c oxidase subunit 1 gene (COI) was amplified using the primers COIintF–COIintR (Casiraghi et al. 2001), and Pr-a–Pr-b (Nakano et al. 2006). PCR conditions were 1 min at 94 °C, followed by 35 cycles at 94 °C for 1 min, 43 °C for 1 min and 72 °C for 2 min, and an extension for 7 min at 72 °C.

All positive PCR products were treated with Exo-SAP-IT (Thermo Scientific), according to the manufacturer’s instructions, and sequenced at the sequencing facility of the Instituto de Biología, UNAM. Consensus sequences were assembled, and base-calling differences were resolved using Geneious Prime v.11. A standard nucleotide similarity search was performed using the BLAST tool with the obtained sequences against the NCBI database. All obtained sequences were deposited at GenBank.

#### Phylogenetic analysis

Phylogenetic relationships of the adult parasites recovered during the necropsy were inferred using the COI sequences. Alignments were performed using MUSCLE (Edgar 2004) through the EMBL-EBI web interface (Madeira et al. 2024). Most appropriate evolutionary model was inferred following the Akaike information criterion (AICc) in MEGA v.11 (Tamura et al. 2021). The TN93 + I + G substitution model was the best model for the data sets analyzed. Phylogenetic trees were constructed using the neighbor-joining algorithm, with 1000 bootstrap replicates in MEGA. Genetic pairwise distances were estimated also in MEGA, using the best model obtained for each sequence set.

## Results

**Fig. 2:**
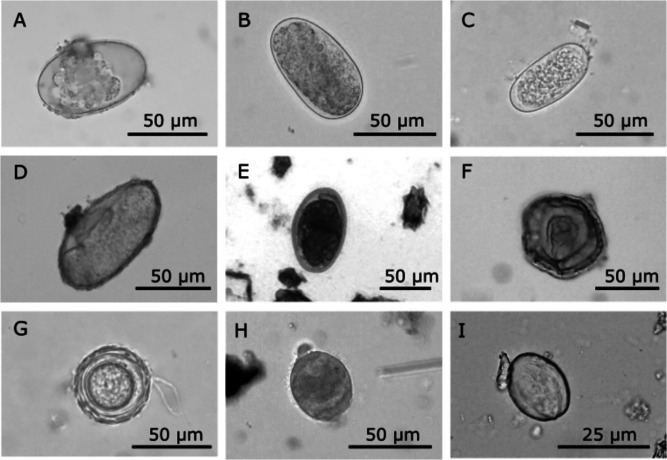
Gastrointestinal parasites identified in deer from southeastern Mexico. **A** Strongylid egg type 1, **B** strongylid egg type 1 in a different developmental stage, **C** *Strongyloides* sp., **D** Strongylid egg type 2, **E ***Mammomonogamus* sp., **F** Anoplocephalid, **G** Ascarid, **H** Ciliate, **I** *Giardia intestinalis* cyst

A total of 140 deer fecal samples were collected from 12 localities in the YP (Fig. 1), including the two captive populations: 97 for white-tailed deer, 33 for Yucatan brocket deer, and ten samples for red brocket deer. We recorded 11 parasite taxa infecting these species including three platyhelminths (two trematodes and one cestode), six nematodes (including two different morphotypes of strongylid eggs), and two protozoans (Table 2, Fig. 2). The white-tailed deer showed the highest parasite richness with 11 taxa, nine of which were also observed in the Yucatan brocket deer. For the red brocket deer, only two samples were found positive, probably due to the small sample size for this host species. Prevalences of infection for all gastrointestinal parasite taxa were low in the three deer species, with nematodes showing the highest prevalence values, followed by protozoans and flatworms.

**Table 2:** Parasite taxa found in deer hosts, the white-tailed deer (*Odocoileus virginianus*), the Yucatan brocket deer (*O. pandora*) and the red brocket deer (*Mazama temama*), and regions (northwestern = NW, north-central = NC, southwestern = SW) across the Yucatán Peninsula Mexico. Percentage of infection followed by density (range of egg/g). Diagnostic method: CP = coproparasitoscopic, MQ = qPCR, MN = nested PCR, and NE = necropsy

			Host species			Region			
Parasite group	Parasite taxa	Diagnostic method	*Odocoileus virginianus*	*Odocoileus pandora*	*Mazama temama*	Ex situ	NW	NC	SW
Platyhelminthes	Trematode	CP	1.03 (100)						2.2
	*Paramphistomum* sp.	CP	1.03 (100)	3.03 (100)				8.3	2.2
	Anoplocephalidae gen.sp.	CP	1.03 (400)	3.03 (400–600)	1.0 (300)				6.5
Nematodes	Ascaridae gen. sp.	CP	2.06 (100)	3.03 (200)			3.6	8.3	
	Strongylid MT1	CP	4.1 (100–200)	3.03 (100)		7.7	1.8	8.3	2.2
	*Strongyloides* sp.	CP	6.2 (100)	3.03 (100)			3.6	41.7	
	*Mammomonogamus* sp.	CP	2.06 (100)	9.1 (100–300)				16.7	6.5
	Strongylid MT2	CP, NE	3.09 (100–200)	3.03 (100)			3.57		4.4
	*Setaria* sp.	MQ, NE	18.6	9.1			26.8		13
Protozoos	*Giardia intenstinalis*	CP, MN	5.2 (100–200)	3.03 (100)	1.0 (100)		5.4	8.3	6.5
	Ciliates	CP	2.06 (100–200)				3.6		
Sample size			97	33	10	26	56	12	46
Positive samples (%)			39.2	39.4	20	11.5	24	66.7	41.3
Parasite richness			11	9	2	1	7	6	8
Taxa per sample			1–2	1–2	1	1	1–2	1–2	1–2
Sampled localities			12	9	4	2	4	2	4
*MT1 and MT2* = morphotype 1 and morphotype 2

For the molecular determination of *Giardia* species, six high quality sequences for the 18S gene were obtained including four from white-tailed deer, one from the Yucatan brocket deer, and one from the red brocket deer. All ~ 130 bp sequences were identical to each other (GenBank accession No PV738950). According to the best match in the BLAST search, all sequences corresponded with *Giardia intestinalis* assemblage B, with an identity of 99–100%. Genetic distance between the obtained sequences and those from assemblage A available in GenBank was 3.4%.

Filariid infections were detected through qPCR and confirmed by standard PCR in white-tailed deer and Yucatan brocket deer, but no infection was detected in red brocket deer. Both host species show relatively low prevalence values. Blast results of the 12S gene showed an identity of 90.3% with *Setaria labiatopapillosa*, supporting the genus identity, but not high enough for a confident species designation.

Regarding geographic origin of samples, we found the highest percentage of parasitized hosts in the NC region, followed by the SW and NW. The most frequent parasite in the NW and SW regions was *Setaria* sp., while in the NC *Strongyloides* sp., and *Mammomonogamus* sp. showed the highest prevalence values. The captive populations showed the lowest percentage of infected animals and the lowest parasite richness, with only white-tailed deer samples found positive for parasites.

**Fig. 3:**
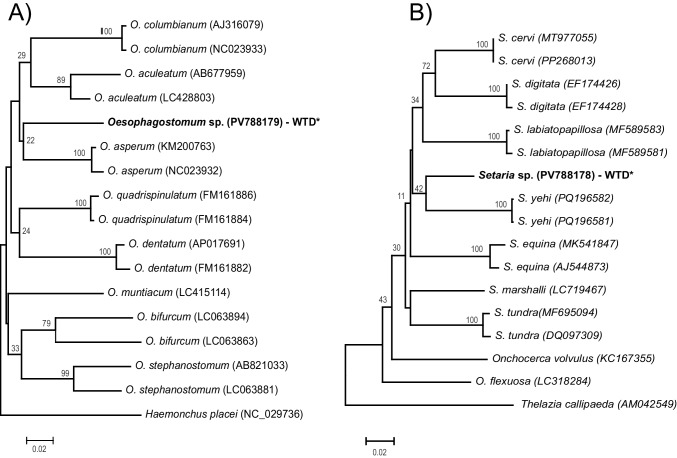
Neighbor-joining phylogenetic analysis of COI gene, using the Tamura-Nei model for the adult parasites of white-tailed deer. **A** *Oesophagostomum* sp., **B** *Setaria* sp. Nodal support indicated by bootstrap percentage. Bold font indicates sequences obtained in the present study. GenBank accession in parenthesis

During necropsy, three nematode specimens were recovered, two strongylids from the gastrointestinal tract, and one filaria from the mesentery. Morphological characteristics of the strongylids corresponded to the description of the genus *Oesophagostomum* such as a reduced buccal capsule divided in an external an internal leaf crown, the presence of a transverse groove near the excretory pore, and a club-shaped esophagus (Negi et al. 2022). Neighbor-joining tree of the ~ 395 bp COI alignment shows these sequences as a sister group of the *O. asperum* clade (Fig. 3A); also, genetic distance with the other *Oesophagostomum* species was on average 14.5% (Table 2). These results suggest that the recovered *Oesophagostomum* corresponds to a different species from those sequenced and available in GenBank.

For the filaria specimen, the 12S sequence was identical to those obtained from the fecal samples, indicating that they belong to the same species (GenBank accession No. PV791783). Neighbor-joining analysis of the ~ 650 bp alignment placed the obtained COI sequence as sister of *S. yehi* (Fig. 3B), corroborating the presence of *Setaria* parasites in these hosts as previously determined by qPCR. Genetic distance with other *Setaria* was on average 10.3% (Table 3).

**Table 3:** Genetic distance (%) of COI gen using the Tamura-Nei model (± sd) between the recovered adult nematodes in white-tailed deer (wtd) and other congeneric species

*Oesophagostomum* sp. – wtd		*Setaria* sp. (wtd)	
*O. aculeatum*	12.4 (0.024)	*S. cervi*	10.2 (0.017)
*O. asperum*	11.8 (0.023)	*S. digitata*	10.9 (0.018)
*O. bifurcum*	18.6 (0.033)	*S. equina*	11.4 (0.019)
*O. columbianum*	14.8 (0.029)	*S. labiatopapillosa*	9.8 (0.016)
*O. dentatum*	15.2 (0.028)	*S. marshalli*	10.3 (0.019)
*O. muntiacum*	15.2 (0.028)	*S. tundra*	9.9 (0.016)
*O. quadrispiculatum*	13.1 (0.026)	*S. yehi*	9.5 (0.016)
*O. stephanostomum*	15.1 (0.028)		

## Discussion

This study reports parasitological information for three species of deer found in southern Mexico, adding to the general knowledge of these species and the host-parasite associations in which they are immersed. Parasite richness was similar among the three sampled regions (NW = 7, NC = 6, SW = 8); however, not all taxa were found across all study sites. Differences in the parasite fauna among regions may reflect geographical variation, including climatic conditions and hosts assemblages specific to each region. Nonetheless, disparities in sample size and host coverage among regions could also account for the observed variation in parasite composition. Captive populations exhibited the lowest parasitic values. These animals are maintained under controlled conditions, receive supplemented diets, and are routinely subjected to veterinary care. Consequently, low levels of parasitism are expected in these populations, which may indicate effective management practices that contribute to deer welfare.

Most of the observed parasite taxa coincide with previous reports for deer in the area and from Mexican populations (see Table 1) (Gallina-Tessaro et al. 2019a; Mora-Collado et al. 2022). The rumen flukes *Paramphistomum liorchis* and *P. cervi* have been reported in deer (Carreno et al. 2001; Cottingham et al. 2022), the latter occurring in white-tailed deer and red brocket deer from Chiapas in southern Mexico (Romero-Castañón et al. 2008). It is highly possible that the *Paramphistomum* eggs observed in the white-tailed deer and the Yucatan brocket deer belong to this species; however, this needs to be further determined with molecular data. The unidentified trematode egg found in white-tailed deer samples resembles those of *Fascioloides magna*; however, this liver fluke has not been reported in deer populations across Mexico, although it has been frequently observed in deer and other ruminants in other areas of North America (Campbell and Vercauteren 2011; Cottingham et al. 2022). These flukes have a complex life cycle involving one intermediate host and two aquatic larval stages, the miracidium and the cercariae; deer can get infected by ingesting metacercaria encysted on the vegetation (Malcicka 2015). Infection with giant liver flukes and rumen flukes tend to be more common in moist sites (Cottingham et al. 2022); this could explain that these parasites were only found in deer populations inhabiting the humid forest regions (NC and SW), and it was not recorded in the dryer tropical forest of NW area.

Cestodes are also commonly reported as parasites of deer; these mammals can serve as definitive host for the anoplocephalid *Moniezia* spp. (Campbell and Vercauteren 2011; Cottingham et al. 2022). The three species of deer were positive for anoplochepalid eggs only in localities of the SW region. Unidentified species of *Moniezia* have been reported in captive white-tailed deer populations in YP (Montes Pérez et al. 1998). As definitive hosts, deer are infected with *Moniezia* by accidentally ingesting mites containing cysticercoids, the parasite larval stage (Cottingham et al. 2022). Since *Moniezia* is the only anoplocephalid reported to infect these hosts, it is possible to suggest that the observed anoplocephalids in this study belong to this genus.

Regarding the nematodes, six taxa were found parasitizing white-tailed deer and Yucatan brocket deer. Nematodes are among the most diverse parasite group infecting this type of hosts with over 40 species reported for white-tailed deer (Walker and Becklund 1970). In Mexico, at least nine taxa of nematodes have been reported in wild and captive populations of white-tailed deer and red brocket deer including *Haemonchus* sp., *Cooperia* sp*.*, *Strongyloides* sp., *Trichuris* sp., *Capillaria* sp., *Bunostomum* sp., *Parascaris*, *Ascaris* sp., and *Mammomonogamus* sp. (Montes Pérez et al. 1998; Romero-Castañón et al. 2008; Mukul-Yerves et al. 2014; Salmorán-Gómez et al. 2019). In most cases, as in the present study, nematodes were not identified up to species level because identifications were solely based on egg morphology. Here, we were able to distinguish two different strongylid morphotypes regarding egg’s size, shape, and color. Given the overall morphological features of each egg morphotype and based on the previous records of nematodes in Mexico, morphotype 1 might be consistent with the genus *Haemonchus*, whereas egg morphotype 2 is consistent with *Oesophagostomum*. The identity of the last one was further corroborated by the comparable morphology with the eggs found in the gravid adult female nematode recovered during the necropsy of the hunted white-tailed deer. Nevertheless, this classification of egg morphotypes should be taken with caution given the similar morphology among eggs of Strongylid nematodes.

*Haemonchus contortus* is a common gastrointestinal parasite of deer and ruminants in North America and has been previously reported in white-tailed deer from the YP (Montes Pérez et al. 1998). *Strongyloides* sp. has been also reported infecting white-tailed deer and brocket deer in wild and captive populations in the YP and Veracruz (Mukul-Yerves et al. 2014; Salmorán-Gómez et al. 2019); the species *S. papillosus* has been reported in white-tailed deer from Florida (Forrester et al. 1974). Both species of nematodes possess a direct life cycle where deer can get infected by the ingestion of food or water contaminated with L3 larvae; also, *Strogyloides* L3 can infect new hosts by penetrating their skin and can be vertically transmitted to fawns via intrauterine transmission or by L3 larvae shed in the milk (Forrester et al. 1974; Cottingham et al. 2022). Furthermore, species of these two genera of nematodes are known to have several impacts on their host health. Deer with haemonchosis can show several symptoms of disease including weakness, anemia, emaciation, and even host death (Campbell and Vercauteren 2011). Strongyloidosis occurs usually in fawns with high intensity of infection causing diarrhea, weight loss and dehydration, and occasionally fawns’ death (Forrester et al. 1974). We found the highest prevalences of *Strongyloides* sp. in the NC region, however, in low densities (egg/g) which could be indicative of a low intensity of infection. Continuous monitoring of parasite infections in this region is needed to fully assess the presence and impact of this parasite among deer in the region.

The nematode *Mammomonogamus* sp. has also been reported in white-tailed deer and brocket deer in the YP (Mukul-Yerves et al. 2014). This nematode might cause infections in the respiratory system, and its effects are well documented in felines, primates, elephants, deer, and other ruminants (Bhandari et al. 2022); however, it also has zoonotic potential. Humans can be accidentally infected with *M. laryngeus* via domestic and wild animals, presenting cough and asthma-like symptoms, although infection in humans is quite uncommon and most often found in tropical environments (Agossou et al. 2021; Bhandari et al. 2022).

*Giardia* cysts were observed in the three species of deer and confirmed as *G. intestinalis* through molecular data. This is a parasite of a wide range of mammalian hosts, for which several genotype assemblages have been recognized showing different levels of host specificity (Monis et al. 2003). Molecular analyses of *G. intestinalis* indicate the presence of assemblage B, which coincides with previous observations in other deer species (Stojecki et al. 2015). Assemblage B has a wide range of diverse hosts and can be found in dogs, cattle, and humans (Ryan and Cacciò 2013), suggesting a possible cross-species transmission among deer humans and domestic animals. We also found a ciliate protozoan in the white-tailed deer samples from the NW region. Rumen ciliates from the genera *Entodinium*, *Eudiplodinium*, and *Epidinum* (Ophryoscolecidae) have been previously reported in white-tailed deer from North America (Dehority et al. 1999). These family of ciliates could be considered as commensals since they participate in several digestive processes of their hosts (Cedrola et al. 2022). To the best of our knowledge, there is no available information regarding protozoan parasites for any of the three species of deer in the country; thus, the records presented here constitute the first reports of ciliates in white-tailed deer from Mexico. Further studies are needed to assess the ciliate diversity in these hosts.

The molecular diagnostic methods employed here were efficient to detect the presence of the abdominal worm *Setaria* sp. (Filarioidea) by screening parasite DNA in deer fecal samples. Filariid infections are not infrequent in these hosts, and usually, they do not cause clinical disease (Campbell and Vercauteren 2011). *Setaria* parasites are transmitted by hematophagous invertebrate vectors; hence, microfilariae are rarely shed in feces and then undetectable through traditional coproparasitologic techniques. The methods presented here could facilitate the study of the deer-filariid associations. Likewise, molecular analyses were very useful for the identification of the three adult nematodes recovered from the examined gastrointestinal tract, confirming *Oesophagostomum* sp. and *Setaria* sp. as parasites of white-tailed deer in Mexico. The relatively low identity scores along with the phylogenetic analysis suggest that these parasites belong to a species other than those sequenced and available at GenBank. This highlights the need for a taxonomic assessment and the enrichment of the available DNA library regarding parasites of Mexican deer species, to improve parasitological diagnosis for captive and wild populations.

Studying the parasite fauna of wild animals through non-invasive sampling is challenging, especially for the determination of parasite species richness and intensity of infection, since results rely on the parasitological elements expelled with feces, such as eggs and larvae which in many cases are highly similar to each other or do not present diagnostic morphological features. Additionally, scarce information regarding the parasitic fauna of many wild hosts also limits the diagnosis. The combination of traditional and molecular techniques has proven to be a comprehensive approach for studying and describing parasitological associations in wildlife populations (Solórzano-García and Pérez-Ponce de León 2017; DeCandia et al. 2018; Rojas et al. 2024). In order to improve and potentiate the usefulness of non-invasive sampling and coprological surveys to monitor wildlife health and parasitological dynamics, it is essential to keep working on enhancing the sensitivity and efficiency of the molecular diagnostic methods. Standardizing a simple and efficient protocol for the obtention of molecular data from eggs shed in feces will make the determination of parasitological fauna of these and other wildlife species more feasible and precise (Christensen et al. 1994; Zarlenga et al. 2001; Harmon et al. 2006).

## Conclusion

Eleven parasite taxa were found to parasitize deer species in southeastern Mexico. Some of these parasites can pose potential risk for the deer, as well as other domestic animals like cattle and rabbits (Parija and Chaudhury 2022). These, in conjunction with other pressures such as habitat loss and poaching, could jeopardize the persistence of the deer species in this geographic region. More studies are needed to monitor the health status of these species in the wild, particularly for the understudied Yucatan brocket deer. Deer are among the largest mammals occurring in the tropical forest of the YP, representing key components of trophic webs and ecosystem dynamics. Also, they are important elements of local people’s daily life given their cultural value. Assessing and describing the parasitic fauna to which wild populations of these species are susceptible is required. These studies will not only increase the knowledge about their ecological interactions but also will allow us to evaluate their health status and to identify potential parasitological threats that these populations and the human communities that surround them might be facing.

## Data Availability

The data that support the findings of this study are available from the corresponding author upon reasonable request. All obtained sequences were deposited at Gen Bank.
